# Association of Postdisaster Depression and Posttraumatic Stress Disorder With Mortality Among Older Disaster Survivors of the 2011 Great East Japan Earthquake and Tsunami

**DOI:** 10.1001/jamanetworkopen.2019.17550

**Published:** 2019-12-13

**Authors:** Xiaoyu Li, Jun Aida, Hiroyuki Hikichi, Katsunori Kondo, Ichiro Kawachi

**Affiliations:** 1 Department of Social and Behavioral Sciences, Harvard T.H. Chan School of Public Health, Boston, Massachusetts; 2 Division of Sleep and Circadian Disorders, Department of Medicine, Brigham and Women’s Hospital, Boston, Massachusetts; 3 Department of International and Community Oral Health, Tohoku University Graduate School of Dentistry, Sendai, Miyagi Prefecture, Japan; 4 Division of Community Medicine and Public Health Practice, School of Public Health, University of Hong Kong, Hong Kong; 5 Center for Preventive Medical Sciences, Chiba University, Chiba, Japan; 6 Center for Gerontology and Social Science, National Center for Geriatrics and Gerontology, Obu, Japan

## Abstract

**Importance:**

Depression and posttraumatic stress disorder (PTSD) represent 2 common mental health sequelae of natural disasters. However, to date, no studies have examined whether postdisaster depression and PTSD are associated with increased risk of mortality among community-dwelling survivors of natural disasters.

**Objective:**

To assess whether postdisaster depression and PTSD were associated with mortality in older disaster survivors.

**Design, Setting, and Participants:**

In this cohort study, prospective data were retrieved from older Japanese adults in Iwanuma City, Miyagi Prefecture, which was directly affected by the 2011 Great East Japan Earthquake and Tsunami. The baseline was established 7 months before the disaster (August 2010), and follow-up surveys were conducted approximately 2.5 years afterward (October 1, 2013, to January 31, 2014). Invitations were mailed to every citizen 65 years or older in Iwanuma City. Mortality data were obtained through March 4, 2017. Data analysis was performed from December 1, 2018, to June 30, 2019.

**Exposures:**

Postdisaster depression (Geriatric Depression Scale Short Form score ≥5) and PTSD (Screening Questionnaire for Disaster Mental Health PTSD subscale score ≥4) were measured in 2013.

**Main Outcomes and Measures:**

Mortality data were obtained by linkage to the national long-term care insurance database. Cox proportional hazards regression models were adjusted for predisaster sociodemographic characteristics, health behaviors, social cohesion, predisaster depression, and disaster experiences.

**Results:**

The response rate for the baseline survey was 59.0% (5058 of 8567 individuals), and the follow-up rate was 82.1% (3594 of 4380 eligible respondents). A total of 2965 individuals (mean [SD] age, 73.4 [6.2] years; 1621 [54.7%] female) participated in the study. The mean (SD) follow-up since the 2013 survey was 3.3 (0.5) years. Overall, 974 (32.8%) reported postdisaster depression and 747 (25.2%) reported PTSD. In adjusted models, depression was associated with more than double the risk of mortality (hazard ratio, 2.29; 95% CI, 1.54-3.42); PTSD was not associated with increased risk of mortality (hazard ratio, 1.10; 95% CI, 0.73-1.64). When evaluating the association of the 4-category comorbid depression and PTSD variable with mortality, survivors with depression only (HR, 2.24; 95% CI, 1.43-3.49) as well as those with comorbid depression and PTSD (HR, 2.54; 95% CI, 1.50-4.27) were at increased risk of death during the follow-up period compared with those with neither depression nor PTSD.

**Conclusions and Relevance:**

Depression, but not PTSD, was associated with mortality during 3.3 years of follow-up among older disaster survivors. These findings suggest that long-term mental health consequences of natural disasters may exist and that treating depression in older survivors of disasters may be beneficial.

## Key Points

**Question:**

Are postdisaster depression and posttraumatic stress disorder associated with all-cause mortality among community-dwelling, older natural disaster survivors?

**Findings:**

In this cohort study of data from 2965 individuals, depression was significantly associated with mortality, whereas posttraumatic stress disorder was not. Comorbid depression and posttraumatic stress disorder were not associated with additional risk of mortality compared with depression only.

**Meaning:**

The findings suggest that disaster recovery and reconstruction efforts should be directed to the prevention, screening, and treatment of postdisaster depression.

## Teaser

This cohort study of community-dwelling survivors of the 2011 Great East Japan Earthquake and Tsunami assesses whether postdisaster depression and posttraumatic stress disorder are associated with mortality.

## Introduction

Depression and posttraumatic stress disorder (PTSD) are among the most common mental disorders and confer a heavy burden for individuals, the health care system, and society. The estimated lifetime prevalence is 16.6% for major depressive disorder and 7.8% for PTSD,^1,2^ and depression and PTSD frequently co-occur.^2^ In addition to the significant mental health burden, studies^3,4,5,6,7,8,9,10,11,12,13^ also suggest that depression and PTSD are associated with elevated risk of cardiovascular diseases, diabetes, functional impairment, lower quality of life, and mortality, mostly in patient, veteran, and general population samples.

Individuals exposed to natural disasters experience an increased risk of mortality.^14^ They also experience an increased risk of depression and PTSD. Empirical evidence suggests that approximately one-quarter of disaster survivors have depression and up to one-third develop PTSD.^15,16^ Moreover, both disorders tend to persist over time.^17^ However, despite the abundance of literature documenting the prevalence and factors associated with mental health problems after disaster exposure,^16,17,18,19,20,21,22^ no studies, to our knowledge, have examined whether depression and PTSD are associated with reduced risk of survival among community-dwelling natural disaster survivors, particularly among older survivors—the population most likely to develop mental health problems after disaster exposure.^23^

We sought to examine the associations among postdisaster depression, PTSD, and all-cause mortality among older survivors of the 2011 Great East Japan Earthquake and Tsunami during 3.3 years of follow-up. We hypothesized that depression and PTSD would each be associated with mortality and that survivors with comorbid depression and PTSD would be at greatest risk of mortality. Our data came from a unique Japanese cohort in which information about the mental health status of community-dwelling residents was available from a baseline survey that predated the 2011 disaster, thereby reducing the possibility of recall bias.

## Methods

### Design, Setting, and Participants

The data for this cohort study originated from a larger national population-based cohort, the Japan Gerontological Evaluation Study (JAGES), which was established in August 2010 (ie, 7 months before the 2011 earthquake and tsunami) to investigate prospectively the factors associated with disability onset among community-dwelling Japanese citizens 65 years or older. The protocol for the current study was reviewed and approved by the human subjects committees of Harvard T.H. Chan School of Public Health, Tohoku University, Nihon Fukushi University, and Chiba University. Participants provided written informed consent. All data were deidentified. This study followed the Strengthening the Reporting of Observational Studies in Epidemiology (STROBE) reporting guideline.

The JAGES cohort represents a collaboration of 10 universities and municipal health authorities across the nation. In Japan, the municipal health authorities maintain accurate registers of all citizens in their catchment areas. Using these registers (*jyumin-hyou*), the JAGES team mailed invitations to 169 215 citizens 65 years or older residing in 31 towns and cities throughout the country. A total of 112 123 citizens responded for a response rate of 66.3%. The baseline survey collected information on participants’ sociodemographic characteristics, health behaviors, social interactions, and mental and physical health status.

Seven months after the baseline survey, the 2011 Great East Japan Earthquake and Tsunami directly affected 1 of the field sites of the JAGES cohort, Iwanuma City in Miyagi Prefecture, located 80 km to the west of the earthquake epicenter. A total of 187 residents died (of a total population of 44 187), 5428 buildings were damaged, and 48% of the Iwanuma land area was inundated.^24^ The research team conducted a follow-up survey among survivors approximately 2.5 years after the disaster (October 1, 2013, to January 31, 2014). The survey inquired about participants’ experiences during and after the disaster. Data analysis was performed from December 1, 2018, to June 30, 2019.

**Figure 1.**
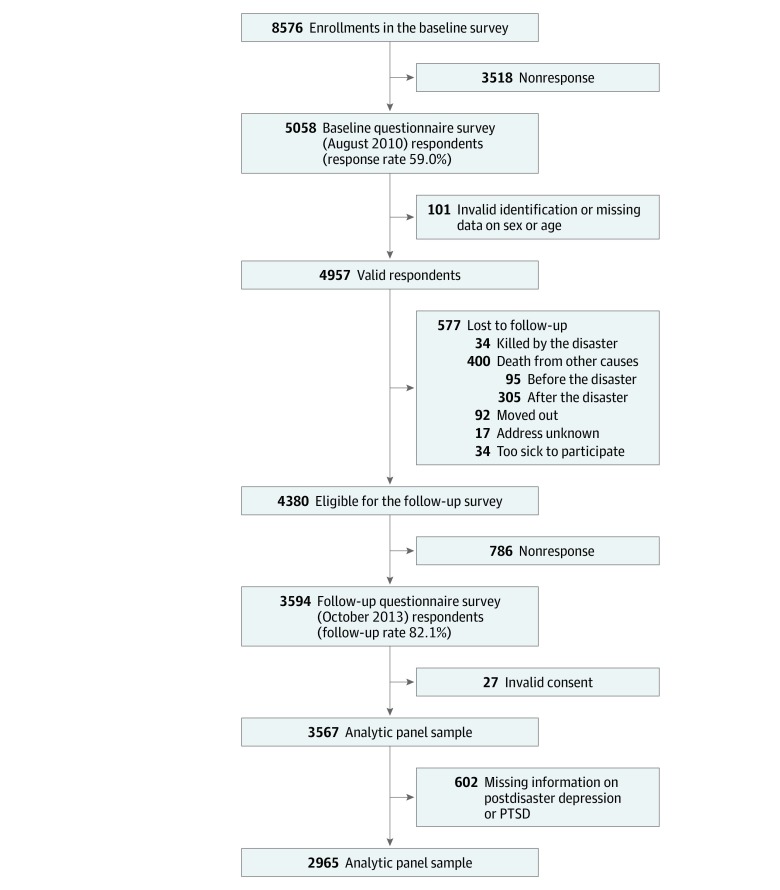
Sample Flowchart PTSD indicates posttraumatic stress disorder.

Figure 1 summarizes the study recruitment and retention. At baseline, a census was undertaken of every resident of Iwanuma City aged 65 years or older. Mortality data until March 4, 2017 (3.3 years after the postdisaster survey), were linked to the Iwanuma cohort.

### Assessment of Postdisaster Depression and PTSD

Depressive symptoms were assessed with the 15-item Geriatric Depression Scale (GDS), which was designed specifically for rating depression in older adults^25^ and translated into Japanese. The GDS score is based on a linear summation of 15 items (eg, “Are you basically satisfied with your life?”), with higher scores indicating worse depressive symptoms. Scores of 5 or higher were considered to be a positive result for depression.^26^ The 15-item GDS has demonstrated strong psychometric properties, with a cutoff point of 5 on the scale having a sensitivity of 92% and a specificity of 81% to detect major depression as ascertained by the Structured Clinical Interview for the *Diagnostic and Statistical Manual of Mental Disorders*, *Third Edition*.^26^

All PTSD symptoms were assessed with the 9-item PTSD subscale of the Screening Questionnaire for Disaster-Related Mental Health (SQD-P), specifically developed and validated for the older Japanese population after the 1995 Kobe earthquake.^23^ The SQD-P score is based on the summation of 9 items (eg, “Do you think about the event when you do not want to?”), with the following predefined categories: slightly affected (score of 0-3), moderately affected (score of 4-5), and severely affected (score of 6-9). Following the approach described in prior literature,^27^ we dichotomized the PTSD scores to enable a simpler interpretation in which the moderately and severely affected (scores of ≥4) were considered to be positive for PTSD. The SQD-P has been psychometrically validated in older Japanese adults affected by disasters and has shown good validity and reliability. The receiver operating characteristic curve was 0.91 for diagnosing PTSD against the Clinician Administered PTSD Scale.^23^

We also created a categorical variable to assess comorbid depression and PTSD. Participants were divided into 4 mutually exclusive groups: those with neither depression nor PTSD, those with depression only, those with PTSD only, and those with both depression and PTSD.

### Assessment of All-Cause Mortality

The main outcome was all-cause mortality. Mortality data for the participants up to March 4, 2017, were obtained from the national long-term care insurance database, which gave a mean (SD) of 3.3 (0.5) years of follow-up after postdisaster depression and PTSD were measured in 2013.

### Covariates

We controlled for predisaster sociodemographic characteristics, health behaviors, social cohesion, predisaster mental health, and disaster experiences, which have been previously reported to be associated with postdisaster mental health disorders and survival.^16,28,29,30,31^ Specifically, adjusted predisaster covariates included age, sex, marital status, household income, education, smoking status, drinking status, body mass index, social cohesion, and depression (measured by the 15-item GDS), all of which were measured at baseline in 2010. Disaster experiences were measured in 2013, which included financial hardship, property damage, health care disruption, and death of close relatives or friends.

### Statistical Analysis

Kaplan-Meier plots and log-rank tests were used to evaluate the cumulative incidence of all-cause mortality according to the presence or absence of depression and PTSD. The primary analyses used Cox proportional hazards regression models to examine the associations of postdisaster depression and PTSD with all-cause mortality. We tested a series of models that sequentially adjusted for potential confounders. Model 1 included only postdisaster depression and PTSD. Model 2 additionally controlled for predisaster covariates, including sociodemographic characteristics, health behaviors, social cohesion, and predisaster depression. Model 3 additionally controlled for disaster experiences. In addition, we constructed Cox proportional hazards regression models with the 4-category comorbid depression and PTSD variable (depression only, PTSD only, and both depression and PTSD, each compared with neither depression nor PTSD), which allowed for a different association between each category and mortality.

In prespecified supplementary analyses, we further examined the association among postdisaster depression, PTSD, and mortality by restricting the analytic sample to the 1818 individuals who were free of depression before the disaster (ie, evaluating the risk of mortality associated with incident depression after the disaster). We also examined models that included baseline comorbidities (eg, hypertension) and self-rated health. Lastly, we tested for potential effect modification by predisaster depression on the associations between postdisaster depression and PTSD all-cause mortality.

All analyses were performed in R, version 3.5.1 (R Project for Statistical Computing) using the survival and the survminer packages. All tests were 2-sided, with a significance level of *P* < .05. The data were analyzed from December 1, 2018, to June 30, 2018.

## Results

Of 8576 individuals who were invited to participate, 5058 responded, yielding a response rate of 59.0%. The response rate to the follow-up survey conducted after the disaster was 82.1% (3594 of 4380 individuals who were eligible). The analyses were limited to 2965 (mean [SD] age, 73.4 [6.2] years; 1621 [54.7%] female; 2101 [72.8%] married) of these respondents after excluding those who provided invalid consent (n = 27) and those who provided no information on postdisaster depression or PTSD in 2013 (n = 602). Descriptive statistics of the analytic sample are presented in Table 1. A total of 695 individuals (23.6%) experienced financial hardship after the disaster, 1742 (58.8%) experienced property damage, 319 (10.8%) experienced health care disruption, and 1120 (37.8%) death of close relatives or friends. In terms of exposures of interest, postdisaster depression was more prevalent (32.8%) compared with PTSD (25.2%). In total, 225 participants died during 3.3 years of follow-up. Compared with those who did not provide postdisaster depression and/or PTSD information or those who were eligible for the follow-up survey but did not respond, the analytic sample was younger, was more likely to be male, was more likely to be married, had higher household income and higher educational attainment, and had lower prevalence of depression before the disaster (eTable 1 in the Supplement).

**Table 1.** Descriptive Traits of Analytic Sample^a^

Variables	Finding (N = 2965)
Predisaster variables	
Age, mean (SD), y	73.4 (6.2)
Sex	
Male	1344 (45.3)
Female	1621 (54.7)
Marital status	
Unmarried	786 (27.2)
Married	2101 (72.8)
Household income, mean (SD)^b^	6.8 (3.0)
Education, y	
<6	41 (1.4)
6-9	940 (32.7)
10-12	1271 (44.2)
≥13	622 (21.6)
Smoking status	
Current	316 (11.5)
Former	780 (28.5)
Never	1645 (60.0)
Drinking status	
Current	1099 (37.8)
Former	103 (3.5)
Never	1705 (58.7)
BMI, mean (SD)	23.5 (3.1)
Social cohesion, mean (SD)^c^	3.8 (0.7)
Predisaster depression^d^	
No	1818 (69.0)
Yes	816 (31.0)
Postdisaster variables	
Financial hardship	
No	2251 (76.4)
Yes	695 (23.6)
Property damage	
Half destroyed or worse	450 (15.4)
Partially destroyed	1292 (44.1)
No damage	1187 (40.5)
Health care disruption	
No	2646 (89.2)
Yes	319 (10.8)
Loss of close relatives or friends	
No	1845 (62.2)
Yes	1120 (37.8)
Depression^d^	
No	1991 (67.2)
Yes	974 (32.8)
PTSD^e^	
No	2218 (74.8)
Yes	747 (25.2)

Abbreviations: BMI, body mass index (calculated as weight in kilograms divided by square of height in meters); PTSD, posttraumatic stress disorder.

^a^ Data are presented as number (percentage) of study participants unless otherwise indicated.

^b^ Household income was rated on a 15-item scale (1 = less than 0.5 million JPY [equivalent to 4586 USD], 15 = 12 million JPY [equivalent to 110 052 USD] or more).

^c^ Social cohesion score ranged from 1 (low) to 5 (high).

^d^ Depression was measured using the Geriatric Depression Scale.

^e^ Posttraumatic stress disorder was measured using the Screening Questionnaire for Disaster-Related Mental Health.

**Figure 2.**
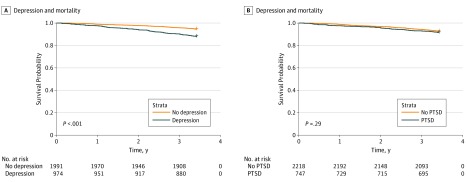
Cumulative Incidence Curves for Postdisaster Depression, Posttraumatic Stress Disorder (PTSD), and Mortality

The Kaplan-Meier plots in Figure 2 show that mortality rates were significantly higher among those with postdisaster depression compared with those without (log-rank test: χ^2^_1_ = 41.5; *P* < 001). Although mortality rates were higher among those with postdisaster PTSD compared with those without, the difference was not statistically significant (χ^2^_1_ =  = 1.1; *P* = .29).

Assumption of proportionality was tested and met. In Cox proportional hazards regression models, postdisaster depression was significantly associated with mortality (hazard ratio [HR], 2.29; 95% CI, 1.54-3.42) after adjusting for predisaster sociodemographic characteristics, health behaviors, social cohesion, depression, and disaster experiences (Table 2).

**Table 2.** Cox Proportional Hazards Regression Models of the Association Between Postdisaster Depression, PTSD, and Mortality

Risk Factor	HR (95% CI)		
	Model 1^a^	Model 2^b^	Model 3^c^
Depression	2.36 (1.80-3.09)^d^	2.22 (1.49-3.30)^d^	2.29 (1.54-3.42)^d^
PTSD	0.91 (0.67-1.23)	0.99 (0.67-1.45)	1.10 (0.73-1.64)

Abbreviations: HR, hazard ratio; PTSD, posttraumatic stress disorder.

^a^ Model 1 included only postdisaster depression and PTSD.

^b^ Model 2 further controlled for predisaster sociodemographic characteristics, lifestyle covariates, social cohesion, and predisaster depression.

^c^ Model 3 further controlled for disaster experiences.

^d^ *P* < .001.

After full adjustment, the association between PTSD and mortality was not statistically significant (HR, 1.10; 95% CI, 0.73-1.64). When evaluating the association of the 4-category comorbid depression and PTSD variable with mortality, survivors with depression only (HR, 2.24; 95% CI, 1.43-3.49) as well as those with comorbid depression and PTSD (HR, 2.54; 95% CI, 1.50-4.27; *P* = .62 for difference in regression coefficients of the 2 groups) were at increased risk of death during the follow-up period compared with those with neither depression nor PTSD. Participants with PTSD only were not at increased risk of death (HR, 1.02; 95% CI, 0.51-2.04).

In supplementary analyses, when restricting the sample to those free of depression before the disaster, incident depression was significantly associated with mortality (HR, 2.16; 95% CI, 1.29-3.61), whereas PTSD was not (HR, 1.01; 95% CI, 0.55-1.88) after adjusting for covariates (eTable 2 in the Supplement). Inclusion of baseline comorbidities and self-rated health as additional covariates did not change the substantive findings. Moreover, there did not seem to be evidence of effect modification by baseline depression on the associations of postdisaster depression and PTSD with mortality. The HRs (95% CIs) for all covariates from regression analyses are presented in eTables 3 through 5 in the Supplement.

## Discussion

This study presents the first empirical investigation, to our knowledge, on the associations of postdisaster depression and PTSD with all-cause mortality among community-dwelling survivors of a major natural disaster. Data came from a prospective cohort of older survivors of the 2011 Great East Japan Earthquake and Tsunami. Depression was significantly associated with an elevated risk of death, whereas PTSD was not associated with death during 3.3 years of follow-up. Participants with comorbid depression and PTSD had no greater risk of death than those with depression only.

Mental disorders might be associated with risk of mortality through biological and behavioral mechanisms. Postdisaster depression and PTSD trigger a chronic stress reaction in response to trauma. A previous study^32^ suggested that prolonged stress reactions are associated with impaired adaption and increased wear and tear on the body. Depression has been linked to a range of neuroendocrine and inflammatory response alterations (eg, dysregulation of the hypothalamic-pituitary-adrenocortical axis and elevated C-reactive protein, interleukin 1, and interleukin 6 levels) in clinical and community samples^33,34^ that might be associated with increased risk of mortality. Another pathway by which mental health disorders might be associated with risk of mortality is through deleterious health behaviors, such as smoking, binge drinking, physical inactivity, and poor diet, which are risk factors for mortality.^35^ Furthermore, not only is emotional distress associated with adverse health outcomes, it could also be associated with patients’ prognosis by affecting medication and treatment adherence and shaping the course of the diseases.^36^

Contrary to our hypotheses, we only observed significant associations between depression and mortality but not between PTSD and mortality. There are some possible explanations for the lack of association between PTSD and mortality. First, depression and PTSD have many overlapping symptoms (eg, sleep disturbances and withdrawal), and the mechanisms by which depression is associated with risk of mortality might overlap with those by which PTSD is associated with risk of mortality, making differentiation difficult. Second, it is also possible that the effect size of the association between PTSD and mortality was small and that the sample size did not give us enough power to detect a significant association. Third, PTSD was assessed approximately 2.5 years after the disaster. By the time we asked about PTSD, some of the most severely affected individuals might have already died. Thus, our estimates might be conservative.

The burden of depression and PTSD in this analytic sample of disaster survivors was largely comparable to that from prior literature, with the sample in our study having a slightly higher prevalence of depression (32.8% vs 25.8%) and a slightly lower prevalence of PTSD (25.2% vs 30.0%-40.0%) than samples in previous review articles.^15,16^ Only a few studies^10,11^ have examined the association among depression, PTSD, and mortality. Our finding that depression, but not PTSD, was associated with all-cause mortality is consistent with 2 prior studies^10,11^ that examined the associations of depression and PTSD with mortality in the context of trauma-exposed populations. Edmondson et al^10^ examined a sample of Hurricane Katrina survivors with end-stage renal diseases recruited from 9 hemodialysis centers in New Orleans, Louisiana, whereas Kinder et al^11^ evaluated a sample of veterans who were primary care patients from 7 Department of Veterans Affairs medical centers. Both studies^10,11^ reported that depression, but not PTSD, was associated with elevated risk of death. Our study builds on this line of work by (1) examining the research question in a cohort of community-dwelling natural disaster survivors, (2) using predisaster depression information collected at baseline that reduced the possibility of recall bias, and (3) focusing on older adults, who are disproportionately affected by disasters. A prior study^37^ on the Iwanuma cohort reported that individuals with predisaster depression experienced elevated risk of mortality on the day of the disaster compared with individuals without depression. Although the mechanism is not clear, severe depression is often associated with psychomotor retardation in older adults, and it is possible that individuals with depression were delayed in evacuating from their homes during the approximately 1-hour interval between the initial earthquake and the arrival of the tsunami. Our findings further showed that persistent postdisaster depression was associated with mortality during 3.3 years of follow-up.

Further etiologic work assessing biological and behavioral explanations for the observed associations between depression and mortality would be helpful to provide guidance for intervention. Future research might also examine whether successful mental health disorder treatment has a positive effect on disaster survivors’ well-being.

### Limitations 

This study has limitations. First, selection bias could be a potential problem because of the 59.0% baseline response rate and the 82.1% follow-up rate. However, prior research has demonstrated that the Iwanuma cohort at baseline was demographically representative of the whole older adult population in Iwanuma City and that those who responded to the follow-up survey did not differ significantly from those who did not respond.^24,38^ Although those who did not provide information on postdisaster depression or PTSD were more likely to have predisaster depression compared with those who provided information on postdisaster depression or PTSD, the results were likely to be biased toward the null in this scenario. Moreover, 305 individuals died between the 2011 disaster and the 2013 survey (Figure 1). These people were likely to be those who were mostly severely affected by the disaster and might have had a higher prevalence of depression and/or PTSD. Data from the baseline survey show that among the 305 individuals who died before the follow-up survey, 49.8% had predisaster depression, whereas 33.5% of the 4380 individuals who were eligible for the follow-up survey had predisaster depression. Although this finding does not directly show the association of the disaster with postdisaster depression and/or PTSD, it is possible that those with predisaster depression might have had a greater risk of adverse reactions to the disaster. In this case, we are likely to be underestimating the associations between the mental health disorders and mortality.

The second limitation is that postdisaster depression and PTSD were assessed approximately 2.5 years after the disaster. As a result, only participants with persistent depression and PTSD were identified. Future research could evaluate the association of short-term postdisaster depression and PTSD with mortality. In addition, longitudinal study designs could be used to examine the duration or chronicity of depression and PTSD necessary to induce toxic effects. Furthermore, although the instrument used to assess PTSD (SDQ-P) appeared to be well aligned with the Clinician Administered PTSD Scale, with a 0.91 receiver operating characteristic curve. It is likely that it better identifies the more common noncases than the less common cases; thus, it may have failed to identify some true PTSD cases. The SDQ-P also does not include items that assess impairment, which could be as important as symptom endorsement in evaluating whether PTSD is associated with clinically important outcomes. However, the PTSD screener is efficient and cost-effective in clinical practice, especially in the aftermath of natural disasters. Although it might miss some true PTSD cases, the study results indicate that scoring above the threshold on the screener is not associated with increased mortality risk, which suggests that screening for depression might have more clinical utility than screening for PTSD symptoms in older disaster survivors. Nonetheless, postdisaster PTSD screening might be important for identifying adverse outcomes (eg, impairments in work or relationships and declining physical health) other than mortality. Moreover, cause of death was not available in our data set. Future research might consider exploring the associations between postdisaster mental health disorders and cause-specific mortality.

A third potential limitation is the generalizability of these results from older adults. Future work is warranted to assess whether the findings are generalizable to populations who are younger and/or populations from other cultures and whether the findings are generalizable to populations exposed to different types of trauma.

## Conclusions

In this study, postdisaster depression, but not PTSD, was associated with all-cause mortality during 3.3 years of follow-up among older disaster survivors of the 2011 Great East Japan Earthquake and Tsunami. The findings suggest that in addition to its mental health burden, the presence of depression should raise practitioner concerns about its physical health sequelae on disaster survivors. The high prevalence of depression in the aftermath of natural disasters and their toxic effects appear to warrant the health care system to allocate appropriate resources for the prevention and treatment of this type of mental distress. For instance, primary care settings that treat populations exposed to natural disasters might enhance screening for depression. Physicians might be more effective if they could recognize and treat the mental disorder early. Furthermore, the findings suggest that disaster survivors with depression should be monitored for the development of adverse health outcomes and be provided with treatment strategies. Disaster recovery and reconstruction efforts directed to the prevention and treatment of postdisaster depression, a modifiable risk factor, might contribute to improved outcomes for disaster survivors.

## Notes

Supplement. **eTable 1.** Comparisons of Predisaster Characteristics: Analytic Sample, Respondents With Missing Postdisaster Depression/PTSD Information, and Nonrespondents in Follow-up **eTable 2.** Cox Proportional Hazards Models of the Association Between Incident Depression, PTSD, and Mortality, Among the Subsample Free of Depression Before the Disaster **eTable 3.** Cox Proportional Hazards Models of the Association Between Postdisaster Depression, PTSD, and Mortality, Full Results **eTable 4.** Cox Proportional Hazards Models of the Association Between Comorbid Postdisaster Depression and PTSD and Mortality, Full Results **eTable 5.** Cox Proportional Hazards Models of the Association Between Incident Depression, PTSD, and Mortality Using, Among the Subsample Free of Depression Before the Disaster, Full Results
