# Supplementary material for: Association of Postdisaster Depression and Posttraumatic Stress Disorder With Mortality Among Older Disaster Survivors of the 2011 Great East Japan Earthquake and Tsunami
Source: JAMA Netw Open. 2019 Dec 13;2(12):e1917550. doi: 10.1001/jamanetworkopen.2019.17550 (PMC6991206; doi:10.1001/jamanetworkopen.2019.17550)
Supplement: Supplement. — eTable 1. Comparisons of Predisaster Characteristics: Analytic Sample, Respondents With Missing Postdisaster Depression/PTSD Information, and Nonrespondents in Follow-up eTable 2. Cox Proportional Hazards Models of the Association Between Incident Depression, PTSD, and Mortality, Among the Subsample Free of Depression Before the Disaster eTable 3. Cox Proportional Hazards Models of the Association Between Postdisaster Depression, PTSD, and Mortality, Full Results eTable 4. Cox Proportional Hazards Models of the Association Between Comorbid Postdisaster Depression and PTSD and Mortality, Full Results eTable 5. Cox Proportional Hazards Models of the Association Between Incident Depression, PTSD, and Mortality Using, Among the Subsample Free of Depression Before the Disaster, Full Results [file jamanetwopen-2-e1917550-s001.pdf]

Supplementary Online Content

Li X, Aida J, Hikichi H, Kondo K, Kawachi I. Association of postdisaster depression and posttraumatic stress disorder with mortality among older disaster survivors of the 2011 Great East Japan Earthquake and Tsunami. *JAMA Netw Open*. 2019;2(12):e1917550. doi:10.1001/jamanetworkopen.2019.17550

eTable 1. Comparisons of Predisaster Characteristics: Analytic Sample, Respondents With Missing Postdisaster Depression/PTSD Information, and Nonrespondents in Follow-up

eTable 2. Cox Proportional Hazards Models of the Association Between Incident Depression, PTSD, and Mortality, Among the Subsample Free of Depression Before the Disaster

eTable 3. Cox Proportional Hazards Models of the Association Between Postdisaster Depression, PTSD, and Mortality, Full Results

eTable 4. Cox Proportional Hazards Models of the Association Between Comorbid Postdisaster Depression and PTSD and Mortality, Full Results

eTable 5. Cox Proportional Hazards Models of the Association Between Incident Depression, PTSD, and Mortality Using, Among the Subsample Free of Depression Before the Disaster, Full Results

This supplementary material has been provided by the authors to give readers additional information about their work.

© 2019 Li X et al. *JAMA Network Open.*

eTable 1. Comparisons of pre-disaster characteristics: analytic sample, respondents with missing postdisaster depression/PTSD information, and nonrespondents in follow-up				
	**Analytic sample (N = 2965)**	**Respondents missing post-disaster depression/PTSD information (N = 602)**	**Nonrespondents in folllow-up (N = 786)**	
	**Mean (SD) / No. (%)**	**Mean (SD) / No. (%)**	**Mean (SD) / No. (%)**	**P-value[d]**
Age (mean (sd)), y	73.4 (6.2)	75.0 (6.6)	76.0 (7.3)	<0.01
Gender (%)				<0.01
Male	1344 (45.3)	208 (34.6)	326 (41.5)	
Female	1621 (54.7)	394 (65.4)	460 (58.5)	
Marital status (%)				<0.01
Unmarried	786 (27.2)	198 (35.5)	278 (37.2)	
Married	2101 (72.8)	359 (64.5)	469 (62.8)	
Household income (mean (sd))[a]	6.8 (3.0)	6.3 (3.4)	6.4 (3.3)	<0.01
Education (%)				<0.01
Less than 6 years	41 (1.4)	6 (1.1)	35 (4.7)	
6-9 years	940 (32.7)	243 (43.8)	277 (37.2)	
10-12 years	1271 (44.2)	215 (38.7)	286 (38.4)	
13 years or more	622 (21.6)	91 (16.4)	146 (19.6)	
Smoking status (%)				0.03
Current	316 (11.5)	50 (9.5)	86 (12.1)	
Used to	780 (28.5)	122 (23.1)	192 (27.1)	
Never	1645 (60.0)	357 (67.5)	430 (60.7)	
Drinking status (%)				<0.01
Current	1099 (37.8)	178 (30.7)	246 (31.9)	
Used to	103 (3.5)	18 (3.1)	35 (4.5)	
Never	1705 (58.7)	383 (66.1)	489 (63.5)	
BMI (mean (sd)), kg/m^2	23.5 (3.1)	23.5 (3.5)	23.4 (3.4)	0.71
Social cohesion (mean (sd))[b]	3.8 (0.7)	3.8 (0.7)	3.6 (0.8)	<0.01
Pre-disaster depression (%)[c]				<0.01
No	1818 (69.0)	272 (61.8)	382 (59.7)	
Yes	816 (31.0)	168 (38.2)	258 (40.3)	
Definition of abbreviations: BMI = body mass index; PTSD = posttraumatic stress disorder.				
[a] Household income was rated on a 15-item scale (1 = less than 0.5 million JPY, 15 = 12 million JPY or more).				
[b] Social cohesion score ranged from 1 (low) to 5 (high).				
[c] Depression was measured using the Geriatric Depression Scale.				
[d] P-value for comparisons of covariates between groups using Chi-square test and one-way ANOVA test to compare categorical and continuous variables, respectively.				

© 2019 Li X et al. *JAMA Network Open.*

eTable 2. Cox proportional hazards models of the association between incident depression, PTSD, and mortality, among the subsample free of depression before the disaster

	Model 1[a]		Model 2[b]		Model 3[c]	
**Fisk factor**	**HR**	**[95% CI]**	**HR**	**[95% CI]**	**HR**	**[95% CI]**
**Incident depression**	**2.86 *** **	**[1.87, 4.35]**	**2.09 ** **	**[1.25, 3.49]**	**2.16 ** **	**[1.29, 3.61]**
**PTSD**	0.76	[0.47, 1.24]	0.88	[0.49, 1.59]	1.01	[0.55, 1.88]

Definition of abbreviations: PTSD = posttraumatic stress disorder; HR = hazard ratio; CI = confidence interval.
*** p < 0.001; ** p < 0.01; * p < 0.05.
[a] Model 1 included only post-disaster depression and PTSD.
[b] Model 2 further controlled for pre-disaster socio-demographics, lifestyle covariates, and social cohesion.
[c] Model 3 further controlled for disaster experiences.

© 2019 Li X et al. *JAMA Network Open.*

**eTable 3. Cox proportional hazards models of the association between postdisaster depression, PTSD, and mortality, full results**						
	**Model 1[a]**		**Model 2[b]**		**Model 3[c]**	
**Fisk factor**	**HR**	**[95% CI]**	**HR**	**[95% CI]**	**HR**	**[95% CI]**
**Depression**	**2.36 *****	**[1.80, 3.09]**	**2.22 *****	**[1.49, 3.30]**	**2.29 *****	**[1.54, 3.42]**
**PTSD**	0.91	[0.67, 1.23]	0.99	[0.67, 1.45]	1.10	[0.73, 1.64]
Age, years			**1.09 *****	**[1.07, 1.12]**	**1.09 *****	**[1.06, 1.12]**
Female (vs. male)			0.75	[0.44, 1.29]	0.73	[0.43, 1.25]
Married (vs. no)			0.91	[0.61, 1.37]	0.94	[0.63, 1.41]
Household income			1.03	[0.98, 1.09]	1.03	[0.97, 1.09]
Education (vs. 10-12 years)						
<6 years			1.11	[0.38, 3.21]	1.21	[0.42, 3.53]
6-9 years			**1.56 ***	**[1.07, 2.29]**	**1.68 ****	**[1.14, 2.47]**
>12 years			0.92	[0.57, 1.50]	0.94	[0.58, 1.52]
Smoking status (vs. never)						
Current			1.36	[0.72, 2.58]	1.32	[0.70, 2.51]
Used to			**1.71 ***	**[1.06, 2.77]**	**1.67 ***	**[1.03, 2.71]**
Drinking status (vs. never)						
Current			0.70	[0.46, 1.07]	0.71	[0.47, 1.08]
Used to			1.52	[0.80, 2.89]	1.56	[0.81, 2.99]
BMI, kg/m^2			0.97	[0.91, 1.02]	0.97	[0.92, 1.03]
Social cohesion			0.97	[0.75, 1.24]	0.98	[0.76, 1.27]
Pre-disaster depression			0.99	[0.67, 1.48]	0.97	[0.65, 1.45]
Financial hardship (vs. no)					0.69	[0.43, 1.12]
Property damage (vs. no)						
Half destroyed or worse					1.12	[0.64, 1.96]
Partially destroyed					0.98	[0.68, 1.40]
Health care disruption (vs. no)					1.34	[0.82, 2.17]
Loss of close relatives/friends (vs. no)					0.74	[0.51, 1.07]

Definition of abbreviations: PTSD = posttraumatic stress disorder; HR = hazard ratio; CI = confidence interval.
*** p < 0.001; ** p < 0.01; * p < 0.05.
[a] Model 1 included only post-disaster depression and PTSD.
[b] Model 2 further controlled for pre-disaster socio-demographics, lifestyle covariates, social cohesion, and pre-disaster depression.
[c] Model 3 further controlled for disaster experiences.

© 2019 Li X et al. *JAMA Network Open.*

**eTable 4. Cox proportional hazards models of the association between comorbid postdisaster depression and PTSD and mortality, full results**						
	**Model 1[a]**		**Model 2[b]**		**Model 3[c]**	
**Fisk factor**	**HR**	**[95% CI]**	**HR**	**[95% CI]**	**HR**	**[95% CI]**
**Comorbid depression and PTSD**						
(vs. neither depression nor PTSD)						
PTSD only	0.96	[0.58, 1.60]	0.93	[0.47, 1.84]	1.02	[0.51, 2.04]
Depression only	**2.41 *****	**[1.77, 3.28]**	**2.17 *****	**[1.40, 3.38]**	**2.24 *****	**[1.43, 3.49]**
PTSD & depression	**2.13 *****	**[1.49, 3.04]**	**2.21 ****	**[1.35, 3.61]**	**2.54 *****	**[1.50, 4.27]**
Age, years			**1.09 *****	**[1.07, 1.12]**	**1.09 *****	**[1.06, 1.12]**
Female (vs. male)			0.75	[0.44, 1.29]	0.73	[0.43, 1.25]
Married (vs. no)			0.91	[0.61, 1.37]	0.94	[0.63, 1.41]
Household income			1.03	[0.98, 1.09]	1.03	[0.97, 1.09]
Education (vs. 10-12 years)						
<6 years			1.11	[0.38, 3.23]	1.22	[0.42, 3.56]
6-9 years			**1.57 ***	**[1.07, 2.29]**	**1.68 ****	**[1.14, 2.48]**
>12 years			0.92	[0.57, 1.50]	0.94	[0.58, 1.52]
Smoking status (vs. never)						
Current			1.37	[0.72, 2.59]	1.33	[0.70, 2.52]
Used to			**1.72 ***	**[1.06, 2.78]**	**1.68 ***	**[1.03, 2.72]**
Drinking status (vs. never)						
Current			0.70	[0.46, 1.07]	0.71	[0.47, 1.08]
Used to			1.52	[0.80, 2.91]	1.57	[0.82, 3.02]
BMI, kg/m^2			0.97	[0.91, 1.02]	0.97	[0.92, 1.03]
Social cohesion			0.97	[0.75, 1.24]	0.98	[0.76, 1.27]
Pre-disaster depression			1.00	[0.67, 1.48]	0.97	[0.65, 1.45]
Financial hardship (vs. no)					0.69	[0.42, 1.12]
Property damage (vs. no)						
Half destroyed or worse					1.12	[0.64, 1.97]
Partially destroyed					0.98	[0.68, 1.40]
Health care disruption (vs. no)					1.33	[0.82, 2.16]
Loss of close relatives/friends (vs. no)					0.74	[0.51, 1.07]

Definition of abbreviations: PTSD = posttraumatic stress disorder; HR = hazard ratio; CI = confidence interval.
*** $p < 0.001$; ** $p < 0.01$; * $p < 0.05$.
[a] Model 1 included only post-disaster depression and PTSD.
[b] Model 2 further controlled for pre-disaster socio-demographics, lifestyle covariates, social cohesion, and pre-disaster depression.
[c] Model 3 further controlled for disaster experiences.

© 2019 Li X et al. *JAMA Network Open.*

eTable 5. Cox proportional hazards models of the association between incident depression, PTSD, and mortality using, among the subsample free of depression before the disaster, full results

	Model 1[a]		Model 2[b]		Model 3[c]	
**Fisk factor**	**HR**	**[95% CI]**	**HR**	**[95% CI]**	**HR**	**[95% CI]**
**Depression**	**2.86 *****	**[1.87, 4.35]**	**2.09 ****	**[1.25, 3.49]**	**2.16 ****	**[1.29, 3.61]**
**PTSD**	0.76	[0.47, 1.24]	0.88	[0.49, 1.59]	1.01	[0.55, 1.88]
Age, years			**1.09 *****	**[1.05, 1.13]**	**1.09 *****	**[1.05, 1.13]**
Female (vs. male)			0.70	[0.33, 1.48]	0.69	[0.33, 1.46]
Married (vs. no)			0.95	[0.54, 1.69]	0.96	[0.54, 1.70]
Household income			1.01	[0.93, 1.09]	1.00	[0.93, 1.08]
Education (vs. 10-12 years)						
<6 years			1.02	[0.13, 7.73]	0.97	[0.13, 7.39]
6-9 years			1.25	[0.74, 2.09]	1.30	[0.77, 2.19]
>12 years			0.96	[0.54, 1.72]	0.97	[0.54, 1.73]
Smoking status (vs. never)						
Current			1.23	[0.54, 2.79]	1.25	[0.55, 2.84]
Used to			1.47	[0.78, 2.79]	1.48	[0.78, 2.81]
Drinking status (vs. never)						
Current			0.88	[0.51, 1.51]	0.86	[0.50, 1.49]
Used to			1.38	[0.46, 4.08]	1.40	[0.47, 4.20]
BMI, kg/m^2			1.00	[0.93, 1.08]	1.00	[0.93, 1.08]
Social cohesion			1.08	[0.74, 1.57]	1.10	[0.76, 1.61]
Financial hardship (vs. no)					0.59	[0.28, 1.24]
Property damage (vs. no)						
Half destroyed or worse					1.17	[0.55, 2.50]
Partially destroyed					0.90	[0.56, 1.45]
Health care disruption (vs. no)					0.90	[0.41, 2.00]
Loss of close relatives/friends (vs. no)					0.92	[0.57, 1.48]

Definition of abbreviations: PTSD = posttraumatic stress disorder; HR = hazard ratio; CI = confidence interval.
*** p < 0.001; ** p < 0.01; * p < 0.05.
[a] Model 1 included only post-disaster depression and PTSD.
[b] Model 2 further controlled for pre-disaster socio-demographics, lifestyle covariates, and social cohesion.
[c] Model 3 further controlled for disaster experiences.

© 2019 Li X et al. *JAMA Network Open.*
